# Analysis of Surface Roughness and Strain Durability of Eyeglasses Frames by the 3D Printing Technology

**DOI:** 10.1021/acsomega.4c10592

**Published:** 2025-03-24

**Authors:** Burak Malik Kaya, Celal Asici

**Affiliations:** † Vocational School of Health Service, Eskisehir Osmangazi University, Eskisehir, 26040, Turkiye; ‡ Faculty of Science, Department of Physics, Eskisehir Osmangazi University, Eskisehir, 26040, Turkiye

## Abstract

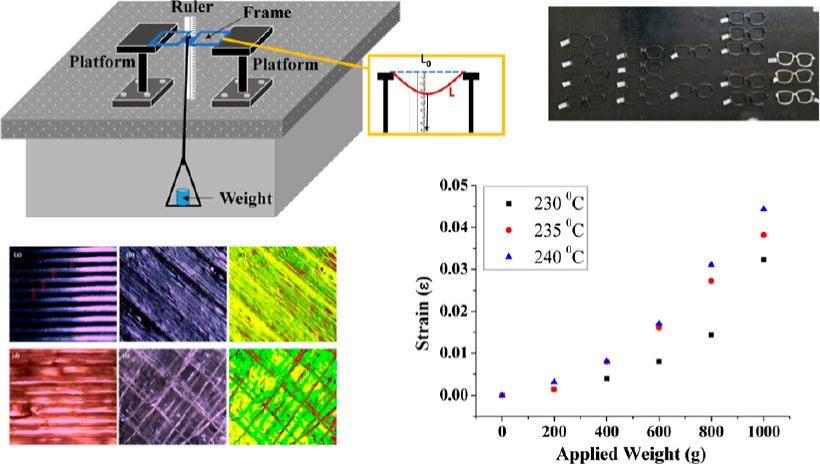

Three-dimensional (3D) printer technology has developed rapidly in recent years and therefore has become the focus of attention in many areas. It has begun to be widely used in many areas in industry, medicine, biomedical, engineering, basic sciences, etc. Among these areas, the optician sector has also widely used 3D technology. Offering personalized eyeglass frame design, freedom of color, shape, and size in frames, 3D technology offers many advantages and conveniences for users and manufacturers. In this project, a 3D printer with high precision and consistency was developed, and eyeglass frames were designed and produced using acrylonitrile butadiene styrene (ABS) and polyethylene terephthalate glycol (PETG) filament types, different printing temperatures, and layer thicknesses. The surface roughness and the durability of the frames were analyzed by using an optical microscope and performing bending tests, respectively. It was observed that the lowest roughness occurred in the ABS-printed frame with 0.20 mm layer thickness at 240 °C temperature, and the highest durability of 54.7 mε obtained with the ABS-printed frames fabricated with 0.20 mm layer thickness at 235 °C temperature. Average roughness (*R*_a_), root-mean-square roughness (*R*_q_), and maximum height of profile (*R*_*z*_) parameters were obtained to analyze surface roughness with respect to temperature change for fabricated frames using ABS and PETG filaments. Thus, the study proves that the production and optimization of customized eyeglass frames can be used not only for commercial and educational purposes in optical stores and optician programs at universities but also in industry, engineering, and daily life purposes.

## Introduction

Three-dimensional (3D) printing, also known as additive manufacturing, is based on the principle of layer-by-layer production, where materials are progressively deposited on top of each other. This technology can be used to quickly produce components with any complex shape by accurately depositing material using solid modeling, based on a computer-aided design (CAD) model or computed tomography (CT) scans under computer control.^1,2^ The 3D printing industry has experienced rapid growth in recent years due to decreasing production costs and improvements in printing precision and speed, which have led to significant advancements in medical equipment, implant materials, and cell printing. The preparation of organ models, rapid production of personalized scaffolds, and direct printing in defective regions can be achieved through 3D printing technology based on a patient’s imaging data, such as CT or magnetic resonance imaging (MRI). This way, 3D printing technology opens up new possibilities for creating bionic tissues or organs, potentially addressing issues such as donor shortages. By adding material layer by layer, 3D printing can convert geometric representations into physical objects.^3^ This 3D process has experienced remarkable growth over the past decade. The commercialization of 3D printing processes began in 1980 with Charles Hull.^4^ Today, 3D printing is widely used in the production of items like artificial heart pumps,^5^ jewelry collections,^6^ 3D-printed corneas,^7^ Paul G. Allen (PGA) rocket engines,^8^ a steel bridge in Amsterdam,^9^ and various products in the aerospace and food industries.^10^

Today, the 3D printing technology has a wide range of application areas, such as robotic technology^11^ to produce fragile and irregular objects, electronics manufacturing^12^ to fabricate circuits, resistors, antennas, etc., aerospace and automotive industries,^13^ especially for functional and end-use parts, and the fashion and eyewear industry,^14−16^ for kinematic dresses, accessories, and unique and functional eyeglasses for luxury customers. 3D printing technology has been extensively preferred and efficiently employed in many sectors due to reduced fabrication costs, freedom in the shape and design of the products, and time-saving advantages. The 3D printing technique is mostly utilized by additive manufacturing (AM) technology, but very recently, multimaterial additive manufacturing (MMAM) technology has been conducted in 3D printing research. These AM and MMAM technologies are well explained in several excellent reviews given in refs (12, 13, 15, 17, and 18). Many AM techniques are capable of integrating multiple materials or properties of a single process. This feature, which is mostly known as MMAM, has the potential to significantly transform current manufacturing and construction practices. In contrast to conventional methods, which typically involve the assembly of single-material components, AM techniques that utilize multiple materials allow for the direct fabrication of objects with varying material properties, obviating the need for subsequent assembly. This approach can enhance efficiency in manufacturing and construction by minimizing the number of production steps and addressing challenges associated with joining disparate materials or components.^12,13,18^

The 3D printing process stems from the layer-by-layer production technology of 3D structures directly from CAD drawings.^19^ 3D printing has emerged as an innovative and versatile technology platform, opening new opportunities and providing many possibilities for companies seeking to increase manufacturing efficiency. Traditional materials such as thermoplastics, ceramics, graphene-based materials, and metals can now be printed using 3D printing technology.^20^ This technology has the potential to revolutionize industries and transform production lines. Adopting 3D printing technology will increase the production speed while reducing costs. At the same time, consumer demand will have a greater impact on manufacturing. Consumers will have more input into the final product and can request customized production according to their preferences. Meanwhile, 3D printing facilities will be closer to the consumer, allowing for a more flexible and responsive production process with enhanced quality control. Moreover, the need for global shipping will decrease significantly with 3D printing. This is because production facilities located closer to the final destination can optimize distribution, saving energy and time with fleet tracking technology. Lastly, adopting 3D printing technology can alter logistics, enabling companies to manage the entire process and offer more comprehensive end-to-end services.^20^ Today, 3D printing is widely used globally. It is increasingly employed in mass customization and the production of various open-source designs across industries such as agriculture, healthcare, automotive, and aerospace.^21^

At the same time, the adoption of 3D printing technology in the manufacturing industry has certain disadvantages. For example, its impact on labor in manufacturing will reduce the need for manual labor, greatly affecting the economies of countries that rely on low-skilled jobs. Furthermore, 3D printing technology allows users to produce various objects, including dangerous items, such as knives, guns, and hazardous materials. Therefore, its use should be restricted to certain individuals to prevent criminals and terrorists from producing weapons undetected. Additionally, individuals who gain access to design files can easily create counterfeit products due to the simplicity of the 3D printing process, which only requires adjusting the data on the machine to produce 3D objects.^22−24^ Besides its application in many fields, 3D technology is also used in optometry. There are various applications, such as contact lens production^25^ and frame production.^15,26−28^

Barbu and Sirbu^29^ fabricated eyeglass frames by using 3D printing technology. They studied a method producing regular frames by utilizing the 3D printing technique to investigate optimum requirements such as the dimensions and shape of the frames, the 3D printer, and system assembling parameters. They modeled the frames using the CAD modeling software so that it can be CATIA, SolidWorks, or any 3D designing software. They employed PLA and ABS filaments at a printing temperature of 210 °C and a 0.15 mm layer thickness, which gives higher quality and resolution for their work. Alam et al.^30^ presented 3D-printed glasses for the color blindness issue. Even though they focused on glass fabrication by using transparent resin, they also fabricated an eyeglass frame. They utilized the SolidWorks design software program to design glass and frame files in stereolithography (STL) format and then converted them to geometric code (G-code) by a slicing tool. The frame was printed at a 50 μm layer thickness, but the printing temperature was not declared. They analyzed the mechanical performance of the lenses and frames by bending and tensile tests. Ayyıldız^28^ studied 3D printing technology to design and fabricate customized spectacles for a 5 years old patient with Goldenhar syndrome. He designed the patient’s midface using surface tomography and designed the frame by utilizing a special software program. He used acrylic resin as filament to fabricate spectacles, which took 14 h production time. The spectacles were washed in an alcohol tank during 1–2 s and then exposed to UV for 24 h. A review study^15^ analyzes several parameters in the 3D printing technology used for eyeglass frames, such as common materials with durability, research methodology, techniques, and design aspects. Studies on 3D printing technology for eyeglass frame production have received interesting attention from researchers, and the results have incredibly high potential for both health and commercial purposes.

In summary, 3D printing technology has emerged as a flexible and powerful tool in the advanced manufacturing industries in recent years. This technology is widely used in many countries, particularly in manufacturing sectors. Therefore, this project provides an overview of 3D printing technologies, their applications, and the materials used in the manufacturing industry. The optimization of a designed 3D printer has been carried out along with the design and production of eyeglass frames with varying temperature, printing speed, and infill settings. Surface roughness was examined using an optical microscope, and average surface roughness (*R*_a_), root-mean-square surface roughness (*R*_q_), and the maximum height of the profile (*R*_*z*_) parameters were analyzed depending on changing temperature. Moreover, strength tests were conducted to measure the durability of the frames produced with ABS and PETG filaments.

## Methodology

A standard 3D printer having features identical to those of conventional 3D printers was designed. The design of the 3D printer was created by using 3D design software such as Solid Edge 2023, FreeCAD, and similar programs. Calibration processes were carried out separately for commercially available ABS and PETG filaments for the project. These calibrations may vary for each filament spool due to various parameters such as filament type, manufacturing method, production date, the ratio of additives and color pigments, and the filament’s dimensional accuracy during production. When these calibrations are not performed, gaps may be created between the layers or walls, significantly reducing the strength of the eyeglass frame. Therefore, these calibrations are extremely important, even if they take a long time.

The design of the eyeglass frame was created by modifying existing eyeglass models using 3D drawing programs like Solid Edge 2023, FreeCAD, and similar software, tailored to suit today’s popular eyeglass frame styles.

Among the most commonly used thermoplastics in today’s 3D printers, PLA (polylactic acid) undoubtedly stands out. Its ability to be printed at relatively low temperatures, combined with its quick cooling properties and lack of undesirable effects such as shrinking or warping during cooling, allows for fast and high-quality prints. However, PLA is not suitable for load-bearing structures like eyeglass frames due to its insufficient durability and tendency to become brittle and break over time when exposed to sunlight and moisture. Especially if left in a car during the summer, the frame would warp due to the heat and become unusable. For this reason, PETG and ABS thermoplastics, other commonly used materials, were utilized in our study. One of the project’s objectives was also to compare the advantages and disadvantages between these two materials.

The effects of the parameters used in the printing of eyeglass frames on characteristics, such as strength, flexibility, and print quality, were investigated. These parameters include the material type, printing temperature, wall thickness, layer thickness, and infill percentage.

The printing temperature must be optimally adjusted for each filament. Not only does the filament type matter, but also factors like the year of production, storage conditions, manufacturer, and the amount of additives, as mentioned earlier, can all lead to variations. To find the optimal printing temperature for the filament used, test prints were conducted at 5.0 °C intervals. If the temperature is too low, then the layers may not bond properly or the material flow may be insufficient, resulting in gaps between the layers and reduced strength. On the other hand, if the temperature is too high, the thermoplastic may lose its properties, burn, or lead to poor print quality.

Wall thickness is another parameter that affects the strength. As the wall thickness increases, strength is expected to improve. However, the thinness of the model limits the number of walls. Setting the wall thickness too high will be ineffective in some models (especially in thin frames), and it will slow the printing process without yielding any significant benefits.

Layer thickness determines how much material is deposited on the vertical axis during printing. A higher layer thickness may hinder bonding between layers, but it can increase strength by adding more material. However, if the layers do not bond properly, cracks and separations may occur between them. Additionally, the printer’s melting capacity and the filament’s maximum flow rate are also factors to consider. If the filament or printer is not suitable for printing thick layers, then the printer will not be able to deposit the required amount of molten thermoplastic, resulting in thinner layers than expected and causing gaps between them. As layer thickness decreases, print quality improves, but overly thin layers can negatively affect strength. Thus, optimizing the layer thickness is crucial. Since conventional printers typically use movement systems with 40 μm accuracy on the vertical axis, prints were taken at 40 μm intervals in this study.

Another important parameter that must be entered into the slicing software used for 3D printers is the infill percentage. This determines the percentage of the space between the print’s walls that will be filled with thermoplastic versus how much will remain hollow. By adjustment of this parameter, print costs and print times can be significantly reduced. However, reducing it too much can negatively affect the print quality and durability. After a certain percentage, no significant improvement is observed in strength, and increasing the percentage raises only costs and print time, so optimizing this parameter is beneficial. Another aspect to consider in infill optimization is the infill pattern. Selecting a pattern suitable for the model being printed can affect the strength. However, since only one frame model was used in this study, the infill pattern was set to “gyroid” and used for all prints.

The printed samples were examined under an optical microscope to obtain images for the layer thickness and surface analysis. Vertical positioning of the frames under the microscope provided 4× magnified images for analyzing layer thickness, while horizontal positioning allowed for surface analysis. Following this, bending and tensile tests were conducted by applying weights at the midpoint of the frames, which were fixed at both ends.

## Results and Discussion

**Figure 1:**
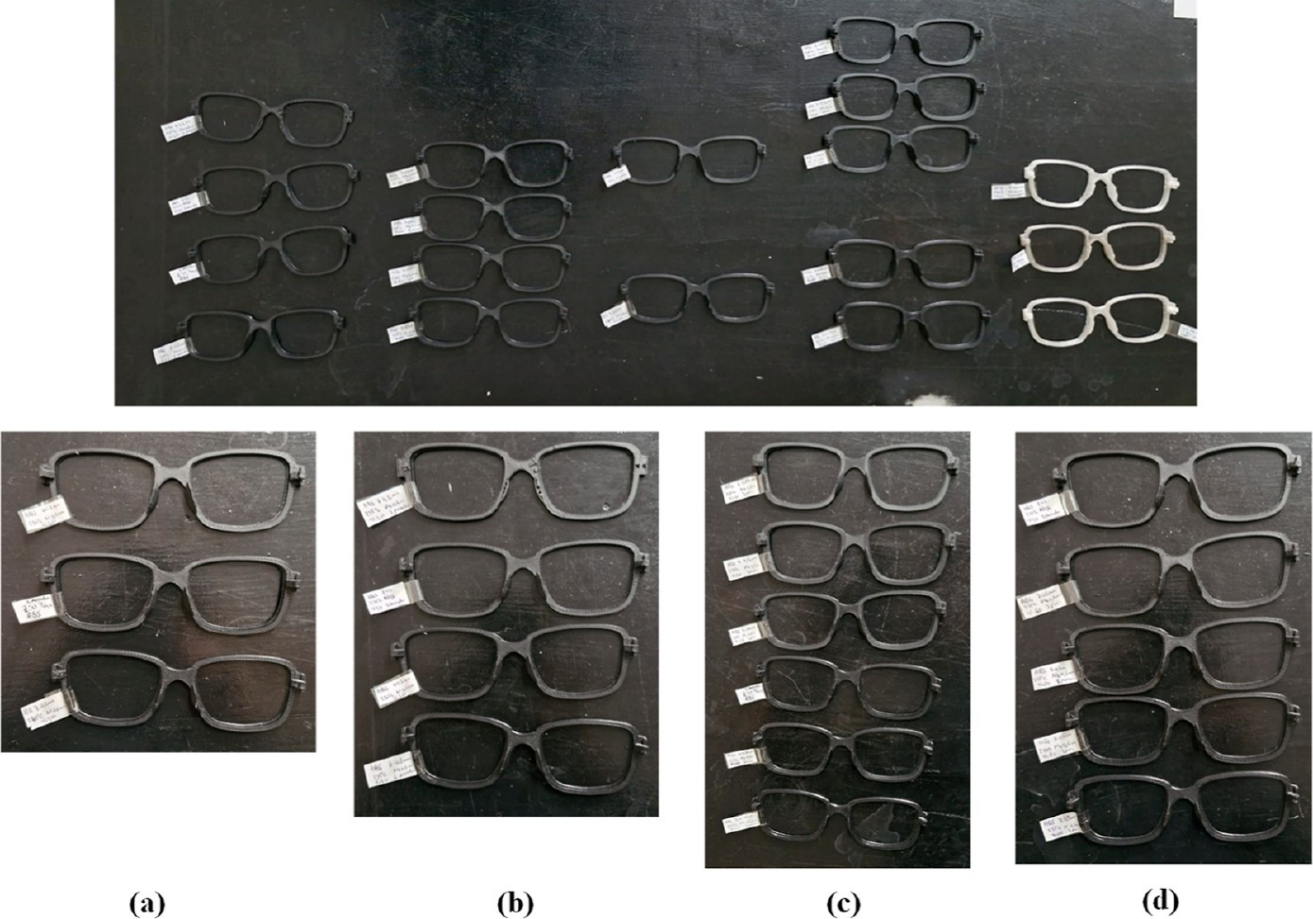
Eyeglass frames printed with different filament types (PETG and ABS) at varying settings: (a) at different production temperatures (230, 235, 240 °C) with 0.20 mm layer thickness and 20% infill, (b) with different infill percentages at 235 °C and 0.20 mm layer thickness, (c) at different layer thicknesses (0.08, 0.12, 0.16, 0.20, 0.24, and 0.28 mm) with 235 °C and 20% infill, and (d) produced at different infill percentages with 235 °C and 0.20 mm layer thickness.

Figure 1 presents eyeglass frames produced by using different types of ABS and PETG filaments at various layer thicknesses and temperatures. Layer thicknesses of 0.16, 0.20, and 0.24 mm were selected, with a temperature range of 230–240 °C. The average production time and cost vary depending on the layer thickness and temperature. The graph showing this variation is provided in detail in the discussion and conclusion sections.

**Figure 2:**
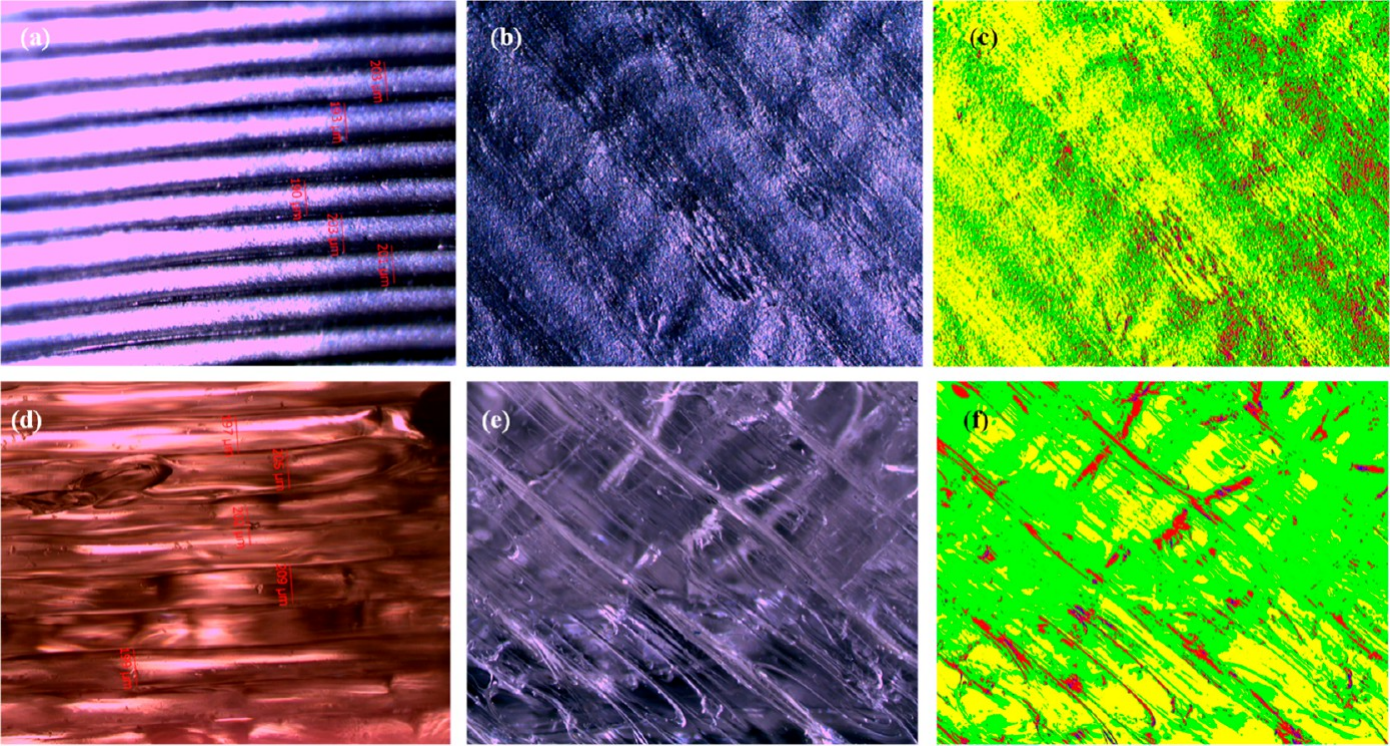
(a–c) Optical microscope and phase images of eyeglass frames produced with the ABS filament at 0.20 mm layer thickness and 230 °C; (d–f) images of frames produced with the PETG filament at the same settings.

Figure 2 displays optical microscope images of eyeglass frames produced using ABS and PETG filaments at a 0.20 mm layer thickness and 230 °C printing temperature. Figure 2a shows a 4× magnified image of a frame, revealing an average layer thickness of 203 μm. Figure 2b, captured at 10× magnification, allows for surface morphology analysis of the frame.

**Figure 3:**
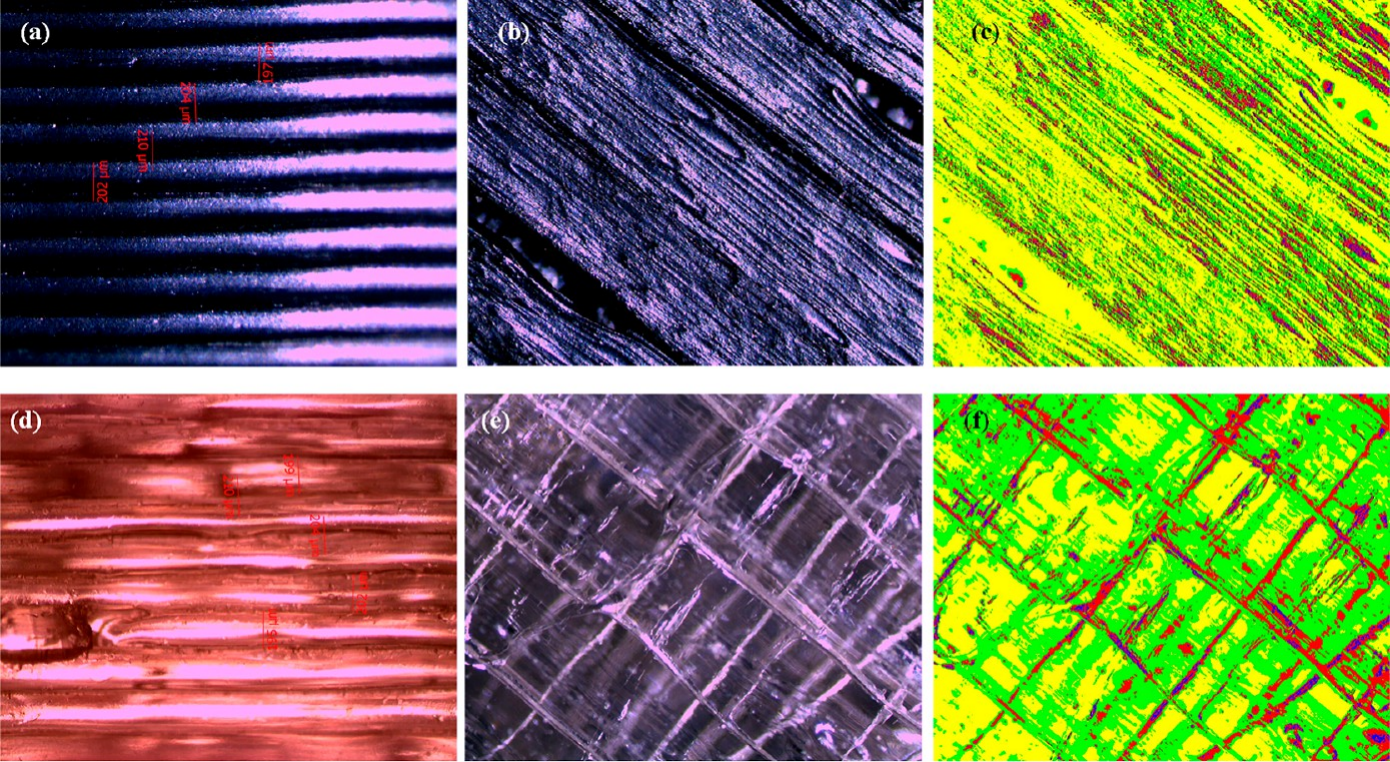
(a–c) Optical microscope and phase images of frames produced with the ABS filament at 0.20 mm layer thickness and 235 °C; (d–f) images of frames produced with the PETG filament at the same settings.

Figure 3 provides optical microscope images of frames printed at a 0.20 mm layer thickness and 235 °C using ABS and PETG filaments. The average layer thickness of the produced frames was determined to be 204 μm. The surface morphology of the frames was examined using 10× magnified optical microscope images, as shown in Figure 3b,d.

**Figure 4:**
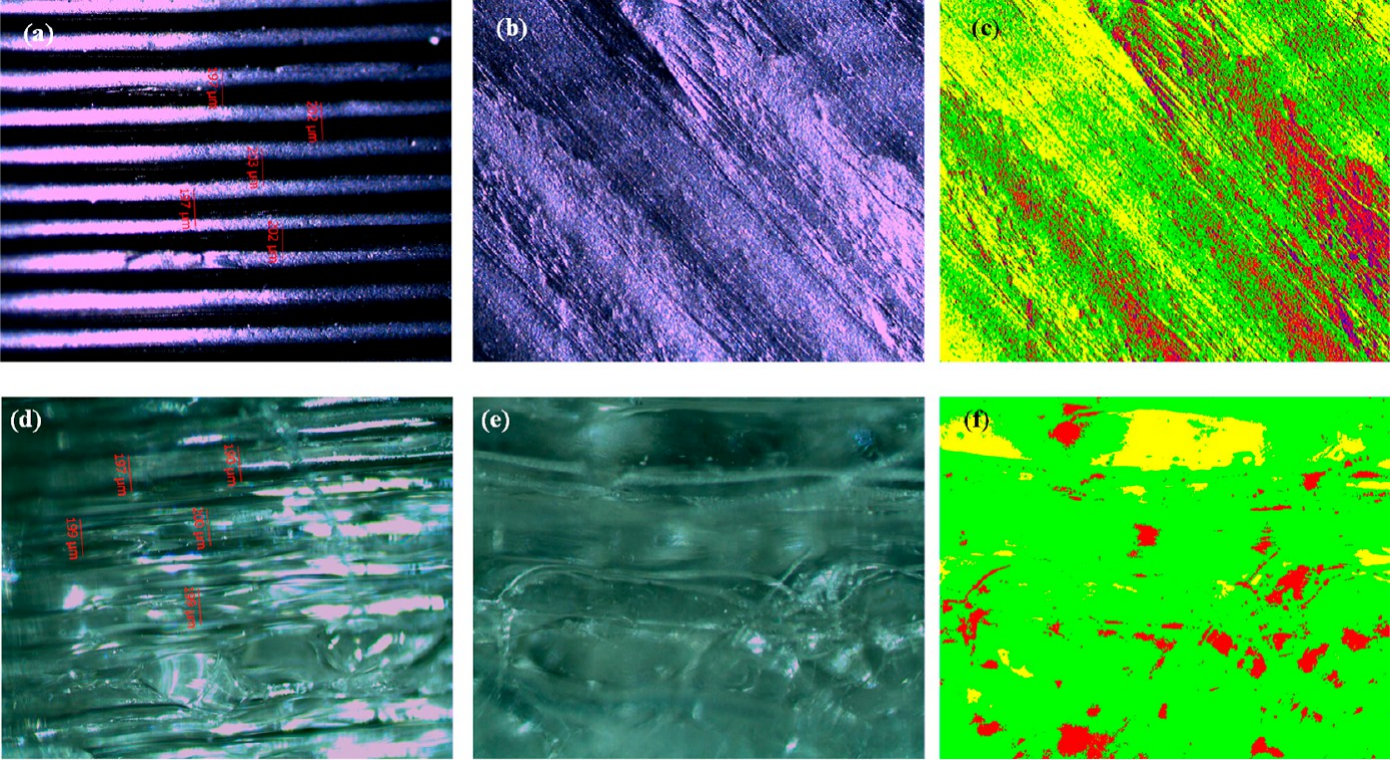
(a–c) Optical microscope and phase images of frames produced with the ABS filament at 0.20 mm layer thickness and 240 °C; (d–f) images of frames produced with the PETG filament at the same settings.

Figure 4 presents optical microscope images of frames printed with ABS and PETG filaments at a layer thickness of 0.20 mm and 240 °C. The average layer thickness was calculated as 200 μm. A 10× magnified image for surface analysis is provided in Figure 4b.

**Figure 5:**
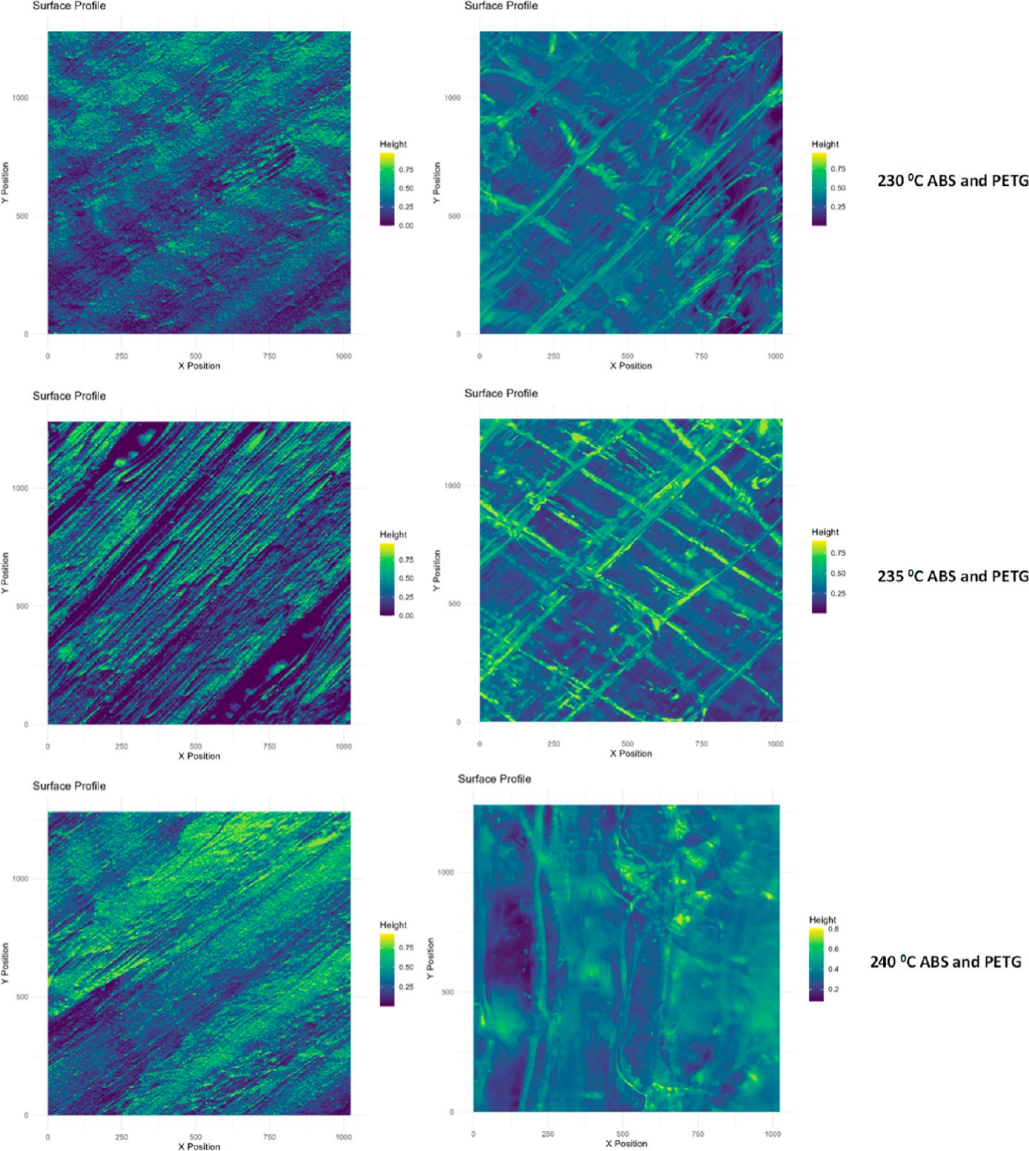
Surface analysis of frames fabricated by ABS and PETG filaments.

Figure 5 shows analyzed figures of frames printed at 230, 235, and 240 °C printing temperature with 0.20 mm layer thickness. The figures were created by using the original images of printed frames with R-code software. The software calculated the surface roughness parameters in terms of pixels. Table 1 was obtained by converting the surface roughness parameters calculated with R-code software.

**Table 1:** Surface Roughness Parameters of Frames Produced with 0.2 mm Layer Thickness at Three Different Temperatures

temperature (°C)	filament type	*R*_a_ (μm)	*R*_q_ (μm)	*R*_*z*_ (μm)
230	ABS	14.58	18.18	97.48
	PETG	9.79	12.97	95.85
235	ABS	18.92	22.84	96.94
	PETG	12.82	16.15	89.52
240	ABS	16.61	20.07	92.48
	PETG	6.63	8.89	72.22

Table 1 shows surface roughness parameters of the frames fabricated with 0.2 mm layer thickness at 230, 235, and 240 °C temperatures. From the table, it is indicated that the average surface roughness (*R*_a_) and the root-mean-square roughness (*R*_q_) of the frame surfaces fabricated by using both ABS and PLA filaments increased and then decreased with increasing temperature. On the other hand, the maximum height of the profile (*R*_*z*_) values gradually decreased for both types with increasing temperature. The surface roughness parameters for a product fabricated by using the ABS filament are comparable with the given studies in refs (31–34) Surface roughness parameters depend on the used filament type, skewness, flow rate, printing speed, and layer thickness, but, on the other hand, perimeter number, infill parameters, and wall thickness do not affect surface roughness.^32,33^ Moreover, the final products can be treated chemically to reduce surface roughness parameters and for preparing effective use.^31^ The relationship between surface roughness and printing temperature can be attributed to various factors involving the material’s extrusion, flow behavior, and solidification during the 3D printing process. At lower temperatures, the ABS filament has no smooth extrusion because its viscosity is higher, resulting in weak layer bonding and high roughness.^35^ Depend on increasing temperature, filament viscosity and extrusion improved, resulting in better layer bonding. The PETG filament used frames have different surface roughness characteristics than ABS used ones. This phenomenon can be explained by several factors related to the material’s properties, extrusion behavior, and the thermal dynamics involved in the printing process. The main factors can be given as improved material flow and viscosity, better layer adhesion, reduced thermal shrinkage and warping, reduced nozzle clogging, and material oozing.^36,37^

**Figure 6:**
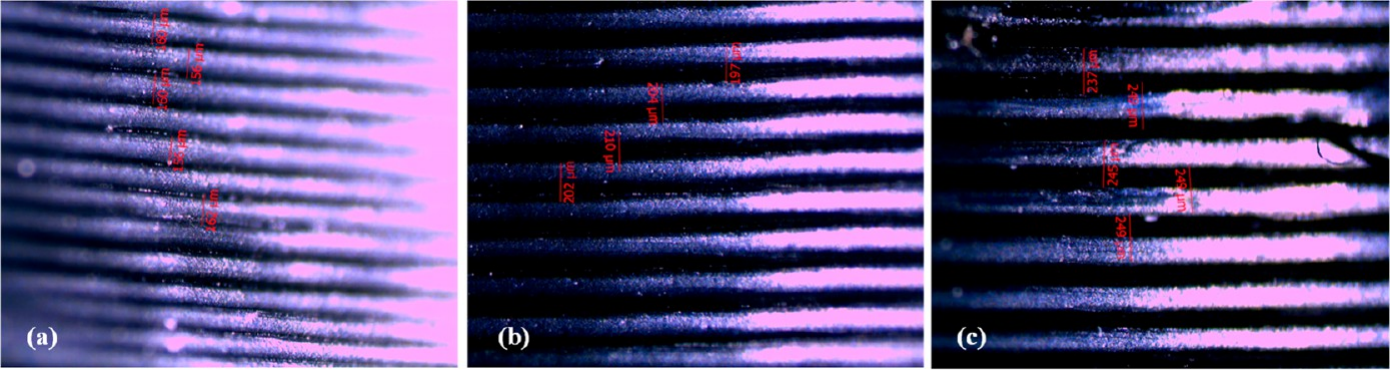
Optical microscope images of frames produced with the ABS filament at different layer thicknesses of (a) 0.12, (b) 0.16, and (c) 0.20 mm, at 230 °C.

Figure 6 shows 4× magnified optical microscope images of frames produced using the ABS filament at different layer thicknesses. Figure 6a–c displays the images of frames produced at 0.12, 0.16, and 0.20 mm layer thicknesses, respectively.

**Figure 7:**
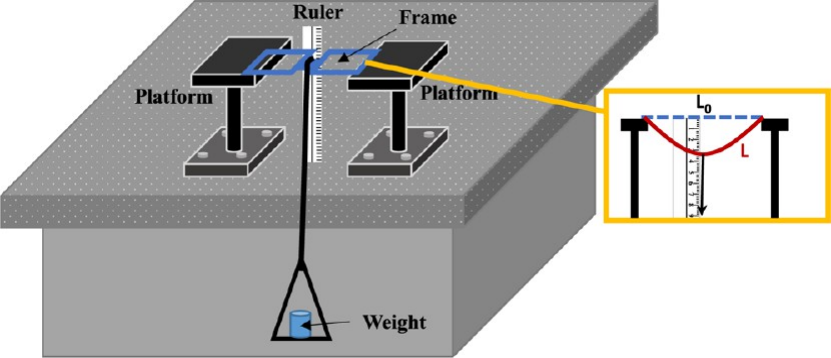
Experimental setup for strain tests of frames.

Additionally, the bending tests and force-strain relationships of the frames produced using ABS and PETG filaments at different temperatures and layer thicknesses were examined. Figure 7 shows the experimental setup of the bending tests. For the bending tests, the frames were horizontally fixed at both ends on the platforms, and weights up to 1000 g were applied in 200 g increments at the midpoint of the bridge section. Before applying the weights, the frames were in an equilibrium position. After applying the weights, the frames were bent down. The changes in length of the frames were calculated by using the frames’ lengths in equilibrium and bending when weights were applied. Strain (ε) was calculated using Δ*L*/*L*_0_ (Δ*L* = *L* – *L*_0_), where *L*_0_ is the length of the frames in equilibrium position and L is the length of frames after applied weights.^38^

The optical microscope images of eyeglass frames produced using PETG and ABS filaments were compared to analyze their layer thicknesses and surface morphologies. Frames were produced at three different temperatures: 230, 235, and 240 °C. The frames produced using the PETG filament with a 0.20 mm layer thickness at these temperatures were analyzed for layer thickness after production, followed by an examination of surface morphology. Similarly, frames produced at 230, 235, and 240 °C with a 0.20 mm layer thickness using a PETG filament were analyzed. Produced frames are shown in Figure 1. Figures 2–4 display the optical microscope images used for layer and surface analysis. According to the analyses, as shown in Figure 2, the average layer thickness of the ABS frame produced at 230 °C with a 0.20 mm layer thickness was found to be 198.00 μm, while for the PETG frame, it was measured at 202.40 μm. At 235 °C with the same layer thickness, the average layer thicknesses for PETG and ABS frames were 203.25 and 202.00 μm, respectively. For frames produced at 240 °C using PETG and ABS filaments, the average layer thicknesses were measured at 199.40 and 197.00 μm, respectively. Considering a margin of error of ±1.50 μm, it was observed that the layer thicknesses of the frames did not change significantly. Since it is known that production temperature affects the degree of bonding between layers, frames were produced at different production temperatures. The most ideal production temperature of 230 °C was determined from tensile and bending tests conducted on the frames. Therefore, as shown in Figure 5, frames were produced using the ABS filament at a production temperature of 230 °C with three different layer thicknesses: 0.16, 0.20, and 0.24 mm. The average layer thickness for frames produced with a 0.16 mm layer thickness was measured at 158.80 μm, while for frames produced with a 0.20 mm layer thickness, it was 203.25 μm, and for frames with a 0.24 mm layer thickness, it was 245.80 μm.

When the surface morphologies of frames produced using ABS filaments were compared, it was observed that surface roughness first increased and then decreased. Phase images provide detailed information about the optical depth structure of the surfaces. By examining the percentages of color distributions on the surface and interpreting what these colors represent, conclusions can be drawn about surface roughness. Yellow represents the lowest points on the surface, while blue represents the highest points. Accordingly, for the frame produced with the ABS filament at 230 °C and a 0.20 mm layer thickness, as shown in Figure 2c, the percentage of color distribution on the surface was as follows: yellow (Y) 49.18%, green (G) 37.46%, red (R) 11.66%, and blue (B) 1.7%. When the PETG filament was used under the same conditions, the percentages were Y: 29.71%, G: 62.53%, R: 7.25%, and B: 0.5%. Comparing these two surfaces, it was found that frames produced with the ABS filament had a rougher surface, while those produced with the PETG filament had smoother surfaces. Surface roughness and strength in 3D-printed products depend on several parameters, including the production speed, filament flow rate, production temperature, layer thickness, and ambient temperature. In this project, production temperature and layer thickness were varied, while other parameters were kept constant for comparison. Similarly, for frames produced with the ABS filament at a production temperature of 235 °C and a layer thickness of 0.20 mm, the color distribution percentages were Y: 56.98%, G: 26.28%, R: 13.5%, and B: 3.24%. For frames produced with the PETG filament under the same parameters, the phase image color distribution percentages were Y: 34.16%, G: 49.95%, R: 13.74%, and B: 2.16%. When the temperature was increased to 240 °C, the color distributions for ABS were Y: 30.92%, G: 43.04%, R: 22.09%, and B: 3.95%, while for PETG, they were Y: 8.11%, G: 85.05%, R: 6.83%, and B: 0.01%. Based on these findings, for frames produced with the ABS filament at a 0.20 mm layer thickness, as the production temperature increased, the percentage of yellow and green areas first increased and then decreased, while the percentage of red and blue areas increased. When analyzing the standard deviations of these changes, they were found to be 22.07% for 230 °C, 23.31% for 235 °C, and 16.45% for 240 °C. This suggests that the frames produced at 240 °C exhibited the lowest surface roughness. For frames produced with the PETG filament, the percentage of yellow, red, and blue areas first increased and then decreased, while the percentage of green areas first decreased and then increased. The standard deviations of these changes were 27.96% for 230, 21.24% for 235, and 40.19% for 240 °C. These results indicate that the frames produced at 235 °C had the lowest surface roughness.

**Figure 8:**
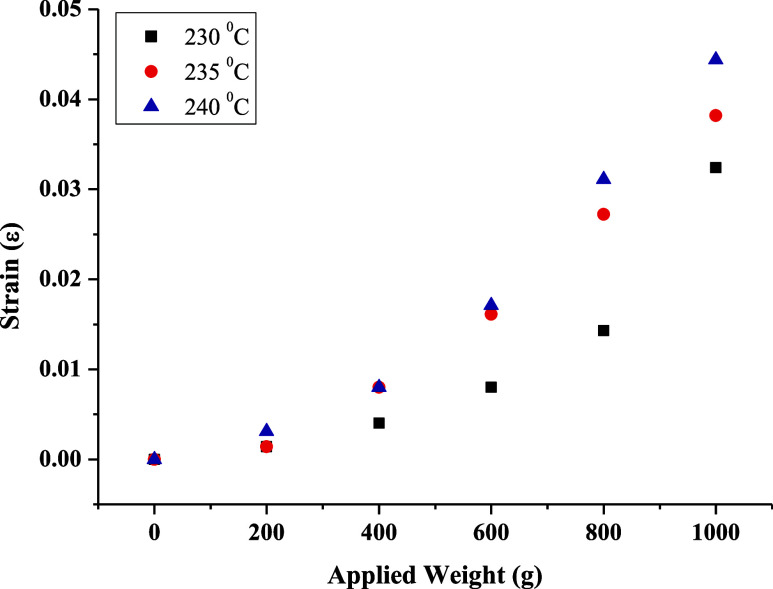
Strain tests of frames produced at different temperatures using the PETG filament with a 0.20 mm layer thickness.

**Figure 9:**
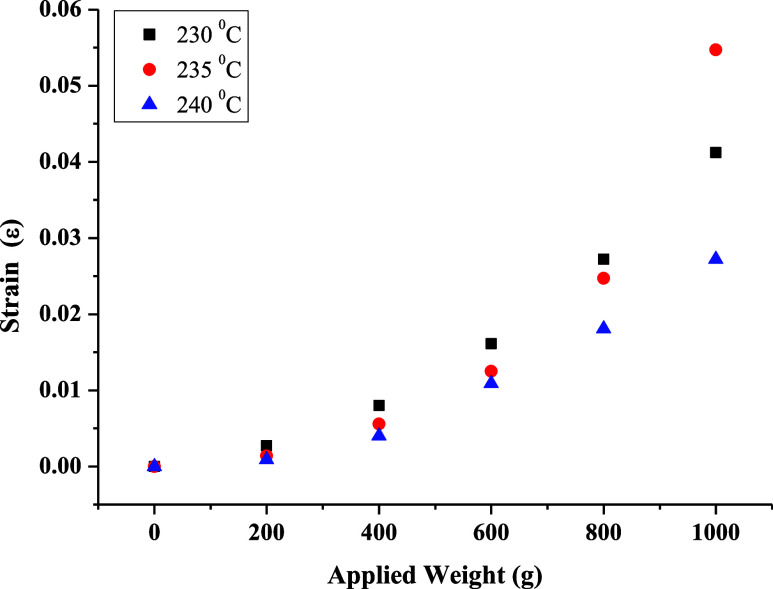
Strain tests of frames produced at different temperatures using the ABS filament with a 0.20 mm layer thickness.

**Figure 10:**
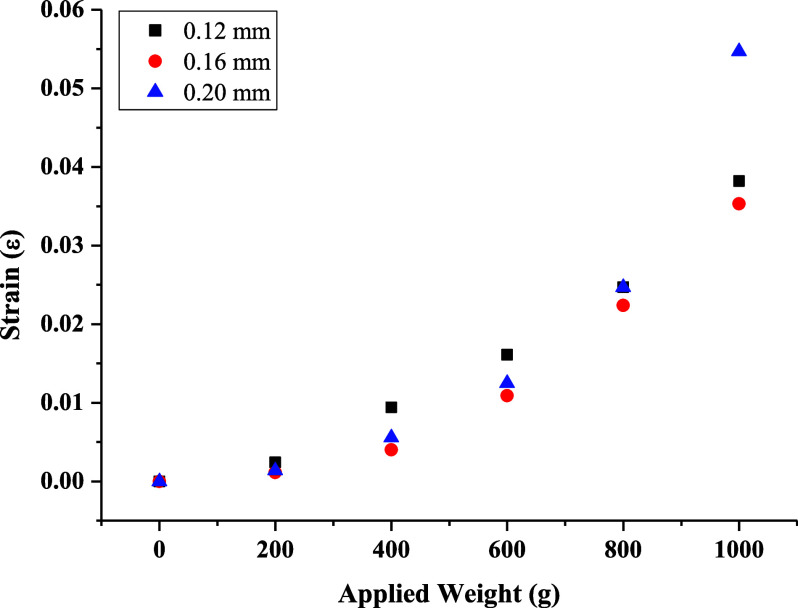
Strain tests of frames produced at different layer thicknesses using the ABS filament at 235 °C.

**Figure 11:**
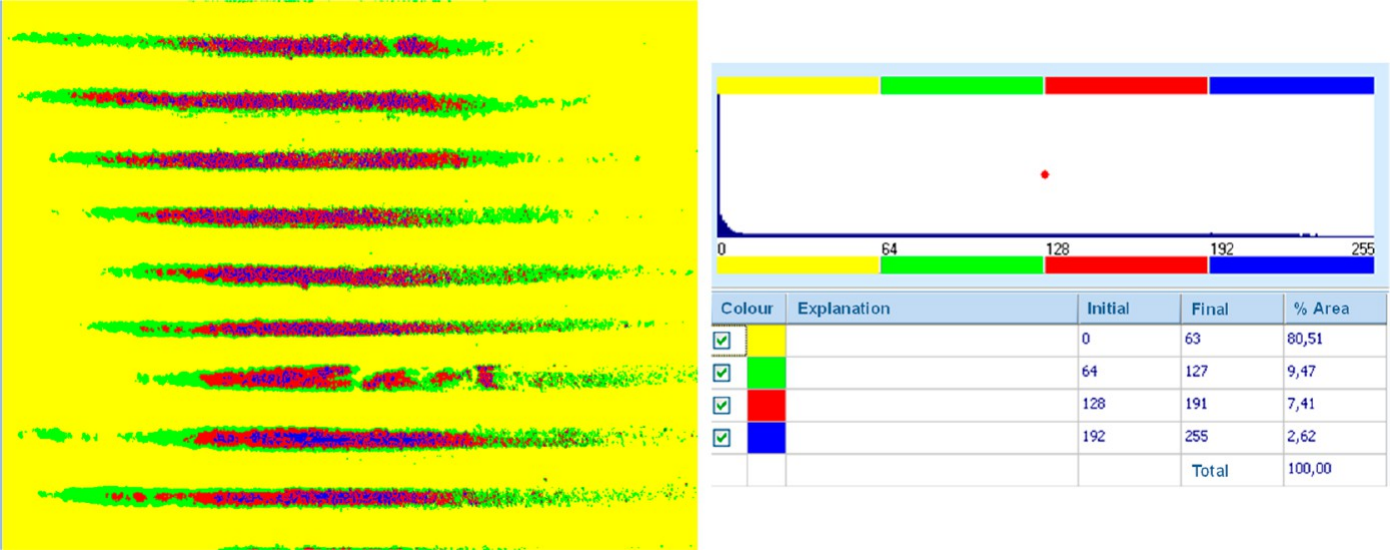
Shows the phase image and color distribution percentage table of the optical image of a frame produced with the ABS filament at 0.20 mm layer thickness and 240 °C.

The strength of the produced eyeglass frames was analyzed by examining the relationship between the applied weight and strain. In Figures 8, 9, and 10, the elongation distance at the midpoint of frames produced using different filaments, production temperatures, and layer thicknesses was related to the applied weights, and durability/flexibility analyses were conducted. The frames were fixed at the edge points, and weights were added to a container suspended from the middle of the bridge section in 200 g increments of up to 1 kg. Weight-strain graphs were obtained by testing frames produced at three different temperatures using PETG and ABS filaments and frames produced using the ABS filament at different layer thicknesses. According to these graphs, the frame with the highest strain coefficient was produced using the ABS filament at 235 °C with a 0.20 mm layer thickness (Figure 11). For frames produced using the PETG filament, the highest strain coefficient was obtained at 240 °C with a 0.20 mm layer thickness. This indicates that frames produced with a 0.20 mm layer thickness are both more durable and more flexible. Figure 10 shows the strain analysis of frames produced at different layer thicknesses at 235 °C. As the layer thickness increases, the strain coefficient first decreases and then increases. This result confirms the expectation that thinner layers have lower strength compared to thicker ones. However, as the layer thickness increases, the bonding between layers decreases, leading to voids, which in turn reduces strength.

Even though 3D printing technology has had an increasing focus since the past decade, it has several disadvantages as much as it has various advantages. 3D printing technology has an outstanding impact on researchers, companies, and customers and has a wide range of application area such as medicine and biomedical,^39^ robotic technology,^11^ electronics,^12^ automotive industry,^40^ and many other applications.^41−43^ It has advantageous features in terms of initial system setup and material costs, software cost, operation, maintenance, customization, personalization, sustainability, and size.^16,44^ The initial cost of a 3D printing system with software and materials may vary depending on requirements such as resolution, volume, compatibility, etc.^10,45^ Several limitations have emerged in terms of material variety, finishing quality, product size, production speed, and durability. Material variety is limited compared to the traditional processes. Plastic and resin materials can be used in 3D printing technology but do not offer high durability and ready-to-use quality. To overcome the deficiencies, researchers have intensively focused on 3D printing technology in many areas.

## Conclusions

Eyeglass frames produced by using ABS and PETG filaments at three different production temperatures (230, 235, and 240 °C) and various layer thicknesses (0.12, 0.16, and 0.20 mm) were analyzed. The layer thicknesses of the frames were measured using an optical microscope, and surface roughness analyses were conducted by examining surface phase maps. Additionally, strain/bending tests were performed by fixing the frames at two edge points and attaching weights at the center of the bridge section. The durability of the frames was analyzed through strain (ε)–weight (g) graphs for the eyeglass frames tested with weights up to 1.0 kg in 200 g increments. Based on the findings, the ideal layer thickness was determined to be 0.20 mm. Surface roughness parameters (*R*_a_, *R*_q_, and *R*_*z*_) were calculated for frames fabricated by using ABS and PETG filaments. For eyeglass frames produced using the ABS filament, the optimal production temperature that resulted in the lowest surface roughness was found to be 240 °C, while for the PETG filament, this temperature was 235 °C. The strain tests also showed that frames produced with ABS and PETG filaments at these respective temperatures demonstrated maximum strain resistance, and the maximum strain value was obtained as 54.7 mε for the ABS printed glasses with a 0.20 mm layer thickness at 235 °C. While all the frames produced with the various parameters were suitable for use, the study focused on identifying the optimal parameters for long-term durability and performance. This research demonstrates that 3D printing technology can be used in the field of optometry to create custom eyeglass frames with the desired layer thickness, production temperature, and filament material. By undergoing basic postprocessing treatments such as vapor smoothing or sanding, the surface roughness of these frames can be significantly reduced. Additionally, coating techniques can be applied to achieve a smooth surface similar to that of commercially produced frames. The rapid development of 3D printing technology in the past decade has made it widely applicable in fields such as healthcare, automotive technology, industry, and education.
